# N-terminal pro-brain natriuretic peptide in a novel screening algorithm for pulmonary arterial hypertension in systemic sclerosis: a case-control study

**DOI:** 10.1186/ar3876

**Published:** 2012-06-12

**Authors:** Vivek Thakkar, Wendy M Stevens, David Prior, Owen A Moore, Jillian Byron, Danny Liew, Karen Patterson, Pravin Hissaria, Janet Roddy, Jane Zochling, Joanne Sahhar, Peter Nash, Kathleen Tymms, David Celermajer, Eli Gabbay, Peter Youssef, Susanna M Proudman, Mandana Nikpour

**Affiliations:** 1 Department of Rheumatology, St Vincent's Hospital Melbourne, 41 Victoria Parade, Fitzroy, Victoria 3065, Australia; 2 Department of Cardiology, St Vincent's Hospital Melbourne, 41 Victoria Parade, Fitzroy, Victoria 3065, Australia; 3 Department of Epidemiology, Biostatistics and Health Research, Royal Melbourne Hospital, Grattan Street, Parkville, Victoria 3050; 4 Institute of Medical and Veterinary Science/SA Pathology, 72 King William Road, North Adelaide, South Australia 5000, Australia; 5 Departments of Clinical Immunology and Immunopathology, Royal Adelaide Hospital, North Terrace, South Australia 5000, Australia; 6 Department of Rheumatology, Royal Perth Hospital, Wellington Street (GPO Box X2213), Perth, Western Australia 6001, Australia; 7 Department of Rheumatology, The Menzies Institute, Private Bag 23, Hobart, Tasmania 7001, Australia; 8 Department of Rheumatology, Monash Medical Centre, 246 Clayton Road, Clayton, Melbourne, Victoria 3168, Australia; 9 Sunshine Coast Rheumatology, PO Box 368, Maroochydore, Sunshine Coast, Queensland 4558, Australia; 10 Canberra Rheumatology, 40 Markus Clarke Street, Canberra, Australian Capital Territory 2601, Australia; 11 Department of Cardiology, Royal Prince Alfred Hospital, Missendon Road, Camperdown, New South Wales 2050, Australia; 12 Advanced Lung Disease Unit and Pulmonary Hypertension Service, Royal Perth Hospital, GPO Box X2213, Perth, WA 6001, Australia; 13 Institute of Rheumatology and Orthopaedics, Royal Prince Alfred Hospital, Queen Elizabeth II Building, Missendon Road, Camperdown, New South Wales 2050, Australia; 14 Department of Rheumatology, Royal Adelaide Hospital, North Terrace, Adelaide, South Australia 5000; 15 The University of Melbourne Department of Medicine, St Vincent's Hospital Melbourne, 41 Victoria Parade, Fitzroy, Victoria 3065, Australia

## Abstract

**Introduction:**

Pulmonary arterial hypertension is a major cause of mortality in systemic sclerosis. N-terminal pro-brain natriuretic peptide (NT-proBNP) has emerged as a candidate biomarker that may enable the early detection of systemic sclerosis-related pulmonary arterial hypertension (SSc-PAH). The objective of our study was to incorporate NT-proBNP into a screening algorithm for SSc-PAH that could potentially replace transthoracic echocardiography (TTE) as a more convenient and less costly "first tier" test.

**Methods:**

NT-proBNP levels were measured in patients from four clinical groups: a group with right heart catheter (RHC)-diagnosed SSc-PAH before commencement of therapy for PAH; a group at high risk of SSc-PAH based on TTE; a group with interstitial lung disease; and systemic sclerosis (SSc) controls with no cardiopulmonary complications. NT-proBNP levels were compared by using ANOVA and correlated with other clinical variables by using simple and multiple linear regression. ROC curve analyses were performed to determine the optimal cut point for NT-proBNP and other clinical variables in prediction of PAH.

**Results:**

NT-proBNP was highest in the PAH group compared with other groups (*P *< 0.0001), and higher in the risk group compared with controls (*P *< 0.0001). NT-proBNP was positively correlated with systolic pulmonary artery pressure (PAP) on TTE (*P *< 0.0001), and mean PAP (*P *= 0.013), pulmonary vascular resistance (*P *= 0.005), and mean right atrial pressure (*P *= 0.006) on RHC. A composite model wherein patients screened positive if NT-proBNP was ≥ 209.8 pg/ml, and/or DLCO_corr _was < 70.3% with FVC/DLCO_corr _≥ 1.82, had a sensitivity of 100% and specificity of 77.8% for SSc-PAH.

**Conclusion:**

We have proposed a screening algorithm for SSc-PAH, incorporating NT-proBNP level and PFTs. This model has high sensitivity and specificity for SSc-PAH and, if positive, should lead to TTE and confirmatory testing for PAH. This screening algorithm must be validated prospectively.

## Introduction

Pulmonary arterial hypertension (PAH) is a major cause of mortality in systemic sclerosis (SSc), accounting for approximately 30% of SSc-related deaths [1,2]. Despite the use of advanced pulmonary vasodilator therapies, SSc-PAH has 1- and 3-year survival rates of 78% and 47%, respectively [3]. In its earliest stages, SSc-PAH is often asymptomatic or minimally symptomatic. Therefore, patients often present late in the natural history of the disease, and more than two thirds are in World Health Organisation functional class (WHO-FC) III and IV at presentation [3-7]. Mounting evidence suggests that earlier detection confers a survival advantage, with a 3-year survival of 70% in those treated in WHO-FC I and II, compared with 3-year survival rates of 50% and 20% in those who are in WHO-FC III or IV, respectively, at diagnosis [3]. In addition, earlier commencement of treatment has been shown to delay the progression of SSc-PAH and lead to improvement in functional class [8,9]. Further, recent evidence has emerged showing that systematic detection programs for SSc-PAH improve the long-term survival of patients when compared with a routine care model that uses signs and symptoms to guide investigations, with an 8-year survival rate of 64% in systematic detection programs compared with only 17% in routine care [10].

Current recommendations from the American College of Cardiology Foundation and American Heart Association (ACCF/AHA), European Society of Cardiology and European Respiratory Society (ESC/ERS), and National Pulmonary Hypertension Centres of the UK and Ireland are for annual transthoracic echocardiography (TTE) in SSc patients, with the latter also recommending measurement of diffusing capacity of the lung for carbon monoxide (DLCO) in patients with limited SSc [11-13]. Collectively, these tests may be expensive, resource and labor intensive, technically challenging, and inconvenient for patients.

The reliance on echocardiography has important limitations. Pulmonary artery systolic pressure (sPAP_TTE_) cannot be measured on echocardiography in 20% to 39% of patients because of absent tricuspid regurgitation and/or insufficient image quality, including up to 29% of patients subsequently found to have pulmonary hypertension at right-heart catheterization (RHC) [14-16]. A wide range exists in the reported sensitivities and specificities for echocardiography in PAH/PH (39% to 100% and 42% to 97%, respectively), as well as an inverse relation between the specificity and sensitivity of echocardiography for identifying patients with PAH/PH-SSc [14]. A study by Hsu *et al*. [17] showed a right ventricular systolic pressure (RVSP) of 47 mm Hg to have a sensitivity of 58% and specificity of 96% for SSc-PAH. Thus, ongoing interest remains in developing methods of noninvasive screening that could improve the sensitivity of current screening methods.

Currently, RHC is the only confirmatory test for PAH. However, because of its invasive nature, RHC is not a feasible screening tool for the SSc population. Therefore, the goal in SSc-PAH detection is to reserve RHC for those patients with a high clinical suggestion of PAH, and to use less-invasive screening tools to risk stratify patients for further assessment with RHC.

Blood biomarkers of PAH, either alone, or in combination with other noninvasive screening investigations, may enable the risk stratification of patients with SSc while potentially reducing the cost and inconvenience of screening for PAH in SSc. N-Terminal pro-brain natriuretic peptide (NT-proBNP) is one candidate biomarker of SSc-PAH. NT-proBNP is a 76-amino acid polypeptide that is released along with BNP, by cardiac myocytes, in response increased ventricular wall stress, as typically occurs with volume overload and ventricular contractile dysfunction [18]. When compared with BNP, NT-proBNP is more sensitive to early increases in sPAP_TTE_, has greater stability as a biomarker, and its assay has good internal validity and reproducibility [19,20]. In a case-control study, Mukherjee *et al*. [20] showed a mean value of NT-proBNP in patients with and without pulmonary arterial hypertension of 3,365 and 347 pg/ml, respectively, with a cut-off value of 395 pg/ml having a sensitivity of 69% and specificity of 100% for identification of SSc-PAH. Allanore *et al*. [21] found that NT-proBNP levels at more than 97% of manufacturer-provided normal levels, particularly when combined with a DLCO/VA < 70%, could predict the development of PAH in eight patients of a cohort of 101 patients over a 36-month period of follow-up.

The objective of our study was to determine the role of NT-proBNP as a screening biomarker for incident SSc-PAH and to evaluate the effect of incorporating this novel biomarker into a screening model for SSc-PAH.

## Materials and methods

### Study population

Patients were selected from the Australian Scleroderma Cohort Study (ASCS). The ASCS is a multicenter study of risk and prognostic factors for cardiopulmonary outcomes in SSc. All patients fulfill either ACR, or Leroy and Medsger criteria for SSc [22,23]. All patients undergo an annual clinical assessment, TTE, and PFT, and have sera collected and stored. Any patient identified by noninvasive screening as having possible PAH (sPAP_TTE _≥ 40 mm Hg, or DLCO ≤ 50% predicted with FVC > 85%, or with DLCO ≥ 20%, or unexplained dyspnea), especially in the presence of symptoms and without adequate explanation on high-resolution CT (HRCT) lung and/or V/Q scanning, are considered for right-heart catheterization (RHC). The ASCS is approved by the human research ethics committees of the 13 participating Australian centers, and patients provide written informed consent at recruitment.

For inclusion in the present study, we selected four groups of patients based on data prospectively recorded in the ASCS database. In group 1, we included 15 consecutive patients with RHC confirmed PAH, based on a mean pulmonary artery pressure (mPAP) ≥ 25 mm Hg and pulmonary capillary wedge pressure (PCWP) ≤ 15 mm Hg [12]. These patients had no more than minor changes of ILD on HRCT.

Group 2 (*n *= 30) consisted of patients who were deemed "at risk" of SSc-PAH on the basis of sPAP_TTE _> 36 mm Hg, and at least one of hemoglobin corrected DLCO (DLCO_corr_) percentage predicted < 50; and/or FVC/DLCO percentage predicted ≥ 1.6 [12,16]. This group included nine patients who underwent RHC because of an sPAP_TTE _> 43 mm Hg, with the finding of "borderline PAH" on RHC (mPAP, 20 to 24 mm Hg, and PCWP, ≤ 15 mm Hg). Group 2 had no evidence of significant obstructive airways disease (forced expiratory volume in 1 second (FEV_1_, liters)/forced vital capacity (FVC, liters) percentage predicted > 0.7) or interstitial lung disease on HRCT lung, and an FVC > 70% predicted.

Group 3 (*n *= 19) consisted of patients with significant ILD, defined as moderate or severe changes of ILD on HRCT, with an FVC < 85% predicted, without evidence of SSc-PAH on RHC (mPAP, < 25 mm Hg, and PCWP, ≤ 15 mm Hg) or TTE (sPAP_TTE_, ≤ 36 mm Hg).

Group 4 (*n *= 30) were SSc controls who did not have evidence of cardiopulmonary complications, based on sPAP_TTE _< 30 mm Hg, normal myocardial function on TTE, DLCO_corr _> 70% predicted, FEV1/FVC percentage predicted > 0.7, no ILD on HRCT (and in those without an HRCT, FVC ≥ 80% predicted), and WHO-FC I or II. All patients selected for groups 2, 3, and 4 were required to have normal RV function assessed semiquantitatively with TTE.

Exclusion criteria for all groups included the presence of abnormal left ventricular systolic or diastolic function for age measured at TTE, abnormal left atrial size, an unrecordable tricuspid regurgitant Doppler signal, and estimated glomerular filtration rate (eGFR) < 30 ml/min.

### Cardiac and pulmonary assessments

Left ventricular systolic and diastolic function was determined by two-dimensional TTE performed within 3 months of collection of serum for the NT-proBNP assay. Systolic PAP was estimated by Doppler TTE (sPAP_TTE_) at rest, based on peak velocity of the tricuspid regurgitant jet and estimation of right atrial pressure of 5 to 10 mm Hg, based on the diameter and respiratory variation of the inferior vena cava. TTE was performed only at tertiary centers for SSc assessment. Pulmonary involvement was assessed with a pulmonary-function test (PFT) and/or HRCT within 3 months of serum collection for the NT-proBNP assay. HRCTs were reported as no, mild, moderate, or severe ILD by a radiologist. All DLCO_corr _(ml/mm Hg/min) values are reported as percentage of predicted values, corrected for hemoglobin [24]. All FEV_1 _(liters), FVC (liters), and FVC/DLCO_corr _values are reported as percentage predicted for sex, race, and height.

### Serum samples and NT-proBNP measurement

All patients had serum collected for NT-proBNP measurement within 3 months of their annual clinical assessment and cardiopulmonary investigations. All PAH patients in group 1 had serum collected for NT-proBNP measurement at the time of their RHC and before the commencement of advanced pulmonary vasodilator therapy. Blood samples were collected at rest into tubes containing EDTA. Samples were centrifuged and stored at -80°C until used. NT-proBNP was measured by using the Elecsys proBNP II sandwich immunoassay on the modular analytics E170 (Roche Diagnostics, Mannheim, Germany). The measurement range of this assay is between 5 pg/ml and 35,000 pg/ml.

### Study design and statistical analysis

In this case-control study, NT-proBNP levels in patients with PAH (group 1) were compared with those of patients considered at "at risk" of PAH (group 2), ILD patients (group 3), and SSc controls (group 4) by using analysis of variance (ANOVA) with Bonferroni multiple test comparison correction. NT-proBNP levels were natural log transformed to satisfy the assumptions of normality and homogeneity of variance.

### Simple linear and multiple linear regression analyses

The correlation between natural log-transformed NT-proBNP and TTE and RHC measures of cardiopulmonary function was quantified by using the Pearson correlation coefficient and simple linear regression. Multiple linear regression models were used to determine the independent correlates of NT-proBNP.

### ROC curve analysis

Receiver operator characteristic (ROC) curve analysis was used for each of four variables (NT-proBNP, DLCO_corr_, FVC/DLCO_corr_, and sPAP_TTE_) to determine the optimal cut point that maximized desired test properties. For each variable, comparison groups were PAH versus controls and PAH versus ILD. For the optimal cut points, results are presented as sensitivity, specificity, and positive and negative likelihood ratios, with 95% confidence intervals (95% CIs). For each variable, area under the curve (AUC) of sensitivity plotted against 1-specificity is also reported with 95% CI.

### Model development and testing

Based on the ROC curve analysis, we created two models for PAH prediction. The first model was based solely on PFT (DLCO_corr _and FVC/DLCO_corr _ratio). The second "composite" model incorporated both NT-proBNP and PFTs. The properties of these models for discriminating PAH from controls, and patients with ILD, were tested by using contingency tables and are presented as sensitivity, specificity, and positive- and negative-likelihood ratios with surrounding 95% CI.

### Application of models to an "at risk" group

The PAH prediction models developed were applied to the at-risk group to determine the proportion of these patients that screened positive.

All statistical analyses were performed by using STATA 11.0 (Statacorp, College Station, TX, USA).

## Results

The clinical characteristics and investigative parameters of patients in each group are shown in Tables 1 and 2.

**Table 1:** Comparison of clinical characteristics of patients in each group

Characteristics	Group 1 PAH	Group 2 At risk	Group 3 ILD	Group 4 Controls	*P *value
Number (*n*)	15	30	19	30	N/A

Age at onset (years)	44.5 ± 12.9	51.5 ± 14.8	40.3 ± 15.6	40.6 ± 13.2	0.015^a^

Age at study (years)	63.3 ± 10.5	66.0 ± 11.8	51.1 ± 12.7	48.7 ± 10.1	< 0.0001^b^

Disease duration (years)	18.8 ± 13.5	14.5 ± 10.4	10.8 ± 7.9	7.8 ± 7.2	0.003^c^

Female, *n *(%)	12 (80)	25 (83)	14 (74)	30 (100)	0.04
Male, *n *(%)	3 (20)	5 (17)	5 (26)	0	

Limited (*n*)	13	28	6	23	< 0.0001^d^
Diffuse (*n*)	2	2	13	7	

ANA, *n*	14	27	17	30	0.43
Anti-Scl70, *n*	1	3	10	5	0.001^e^
Anti-cent, *n*	7	21	0	16	< 0.0001^f^

WHO FC					
-I	0	5	0	25	N/A
-II	2	11	12	5	
-III	11	14	6	0	
-IV	2	0	1	0	

Dis. Duration, disease duration; PAH, pulmonary arterial hypertension; WHO FC, World Health Organisation Functional Class; ANA, anti-nuclear antibody; anti-Scl70, anti- topoisomerase-1 antibody; anti-cent, anti-centromere antibody. ^a^Older age at SSc onset in at-risk group versus other groups. ^b^Older age at time of study in PAH group and at-risk groups versus others. ^c^Longer disease duration at time of study in PAH group versus controls. ^d^Higher proportion of patients with limited subtype in all groups except ILD. ^e^Higher proportion of Scl-70-positive patients in ILD than other groups. ^f^Higher proportion of centromere-positive patients in all groups compared with ILD.

**Table 2:** Comparison of investigative parameters among patients in each group

Investigations	Group 1 PAH	Group 2 At Risk	Group 3 ILD	Group 4 Controls	*P *value
TTE parameters					
TRV (m/s)	3.8 ± 0.7	2.95 ± 0.3	2.54 ± 0.4	2.20 ± 0.2	< 0.0001
sPAP (mm Hg)	65.8 ± 27.3	43.8 ± 7.2	32.1 ± 5.3	26.3 ± 2.6	< 0.0001

RHC results					
mPAP (mm Hg)	40.2 ± 12.5	N/A	N/A	N/A	N/A
mRAP (mm Hg)	10.1 ± 3.3	N/A	N/A	N/A	N/A
PVR (Wood units)	6.2 ± 3.4	N/A	N/A	N/A	N/A

PFT					
FVC (% pred)	75.5 ± 23.4	100.6 ± 19.5	68.7 ± 13.2	102.8 ± 13.4	< 0.0001^a^
DLCO_corr _(% pred)	45.6 ± 11.7	61.0 ± 15.1	48.0 ± 12.7	86.8 ± 13.0	< 0.0001^b^
FVC/DLCO_corr_	1.76 ± 0.38	1.73 ± 0.46	1.46 ± 0.31	1.20 ± 0.20	< 0.0001^c^

6MWD (m)	337 ± 100	N/A	458 ± 84	N/A	N/A

Log NT-proBNP	6.72 ± 1.48	5.18 ± 1.11	4.72 ± 0.57	4.12 ± 0.63	< 0.0001

NT-proBNP (pg/ml)	1818 ± 2367	278 ± 243	133 ± 87	72 ± 38	< 0.0001^d^

6MWD, 6-minute walk distance; DLCO, diffusion capacity of lung for carbon monoxide (% predicted); FVC, forced vital capacity (% predicted); ILD, interstitial lung disease; mPAP, mean pulmonary artery pressure; mRAP, mean right atrial pressure; NT-proBNP, N-terminal pro brain natriuretic peptide; PAH, pulmonary arterial hypertension; PVR, pulmonary vascular resistance; sPAP, systolic pulmonary artery pressure; TRV, tricuspid regurgitant velocity. ^a^Lower FVC in PAH and ILD groups, versus "at risk" and controls

^b^Lower DLCO in PAH and ILD groups, versus "at risk" and controls. ^c^Higher FVC/DLCO in PAH and "at risk" groups, versus ILD and controls. ^d^Higher NT-ProBNP in PAH versus other groups, and "at risk" versus controls.

### Comparison of NT-proBNP levels between groups

**Figure 1:**
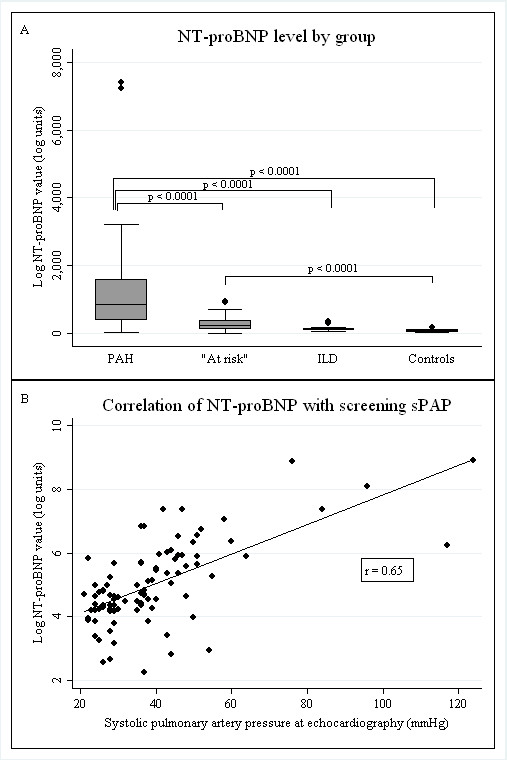
**Comparison of Nt-proBNP level among groups and correlation with sPAP on transthoracic echocardiography**. **(A) **NT-proBNP levels are significantly higher in the PAH group compared with "at risk," ILD, and control groups. Levels in the at-risk group are significantly higher than those in the control group. **(B) **NT-proBNP levels have a significant positive correlation with screening sPAP across all groups. NT-proBNP, N-terminal pro-brain natriuretic peptide; PAH, pulmonary arterial hypertension; ILD, interstitial lung disease; sPAP, systolic pulmonary artery pressure on transthoracic echocardiography (mm Hg); log, natural log; *r*, Pearson coefficient.

NT-proBNP levels were compared between groups (Figure 1A). The PAH group had significantly greater mean NT-proBNP levels compared with the at-risk group (1,817.6 ± 2,367.0 versus 277.6 ± 242.7 ng/ml; *P *< 0.0001), ILD group (133.0 ± 86.6 ng/ml; *P *< 0.0001 versus Group 1), and the control group (72.1 ± 37.8 ng/ml; *P *< 0.0001 versus Group 1). Three patients had NT-proBNP values > 3,000 pg/ml; these patients had the most severe pulmonary hypertension at RHC (mPAP, 58.3 ± 6.0 mm Hg; mean right atrial pressure (mRAP) 13.7 ± 3.5 mm Hg; peripheral vascular resistance (PVR), 11.0 ± 0.9 Wood units). Furthermore, the results from the at-risk group were intermediate to the PAH groups and controls, with a significantly higher mean NT-proBNP level than the controls (277.6 ± 242.7 versus 72.1 ± 37.8 ng/ml; *P *< 0.0001).

### Correlation of NT-proBNP with sPAP_TTE _

A significant positive correlation of NT-proBNP levels with sPAP_TTE _was found in all groups combined (Figure 1B), with a Pearson correlation coefficient of 0.65 (*P *< 0.0001). Simple linear regression comparing NT-proBNP levels in the PAH group and controls showed a significant relation between NT-proBNP and sPAP_TTE _(β = 0.05; 95% CI, 0.04 to 0.06; *P *< 0.0001). As the PAH group were older (63.3 ± 10.5 versus 48.7 ± 10.1 years; *P *= 0.001) and had a longer disease duration (18.8 ± 13.5 versus 7.8 ± 7.2 years; *P *= 0.003) compared with controls, a multivariable model including NT-proBNP, age, disease duration, and sPAP_TTE _was used and showed that the sPAP_TTE _was independently associated with the NT-proBNP level (β = 0.05; 95% CI, 0.03 to 0.06; *P *< 0.001) but not age (*P *= 0.19) or disease duration (*P *= 0.38).

### Correlation of NT-proBNP with cardiopulmonary hemodynamics

**Figure 2:**
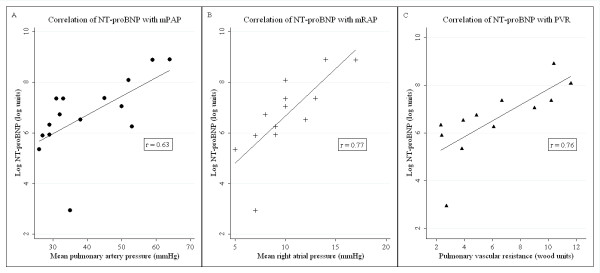
**Correlation of NT-proBNP with RHC parameters**. In patients with PAH, NT-proBNP levels have a significant positive correlation with RHC parameters of **(A) **mPAP; **(B) **mRAP; and **(C) **PVR. NT-proBNP, N-terminal pro-brain natriuretic peptide; mPAP, mean pulmonary artery pressure (mm Hg); mRAP, mean right atrial pressure (mm Hg); PVR, pulmonary vascular resistance (Wood units); PAH, pulmonary arterial hypertension; Log, natural log; *r*, Pearson coefficient.

A significant positive correlation of NT-proBNP with a number of important cardiopulmonary parameters on RHC was found (Figure 2), including mPAP (mm Hg) (correlation coefficient = 0.63; *P *= 0.013), PVR (Wood units; correlation coefficient = 0.76; *P *= 0.005), and mRAP (mm Hg; correlation coefficient = 0.77; *P *= 0.006).

### The effects of comorbid illness and treatment on NT-proBNP levels

In simple linear regression analysis, the use of calcium channel blockers was not associated with NT-proBNP levels in the PAH group (β = -1.33; 95% CI, -2.85 to 0.19; *P *= 0.082). Furthermore, calcium channel blocker use was not associated with NT-proBNP levels in multivariable models that included markers of PAH severity (cardiac output (*P *= 0.273), mRAP (*P *= 0.209), or PVR (*P *= 0.248)). Simple linear regression analysis did not show renal function (*P *= 0.654) or body mass index (*P *= 0.996) to be associated with NT-proBNP levels in the PAH group.

### ROC curve analysis for single variables

As seen in Table 3, ROC curve analysis for each variable was performed for PAH versus controls, and PAH versus ILD. An NT-proBNP cut point of ≥ 209.8 ng/ml for PAH versus controls had a sensitivity of 92.9% and specificity of 100%, with a high AUC of 0.93 for diagnosing PAH. Similarly, an NT-proBNP cut point ≥ 360.5 mg/ml for PAH versus ILD had a sensitivity of 85.7% and specificity of 100%, with an AUC of 0.92 for diagnosing PAH. A DLCO_corr _< 70.3% completely segregated PAH from controls (sensitivity, 100%; specificity, 100%; AUC = 1.0). However, no optimal cut point for DLCO_corr _could be determined that adequately segregated PAH from ILD. An FVC/DLCO_corr _≥ 1.66 had a sensitivity of 64.3% and specificity of 96.7%, with an AUC = 0.90 for PAH versus controls. The FVC/DLCO_corr _was higher at ≥ 1.82 for PAH versus ILD, with a sensitivity of 50.0%, specificity of 94.4%, and an AUC of 0.71 for PAH versus ILD.

**Table 3:** Results of ROC curve analysis and contingency table analysis for NT-proBNP and PFT

Variable	Comparison groups	Optimal cut point	Sensitivity (95% CI)	Specificity (95% CI)	+LR	-**LR**	AUC
NT-proBNP (ng/ml)	PAH versus control	≥ 209.8	92.9% (64.1%-99.6%)	100% (85.9%-99.7%)	-	0.07 (0.01-0.47)	0.93 (0.80-1.0)
	
	PAH versus ILD	≥ 360.5	85.7% (56.2%-97.0%)	100.0% (71.8%-99.5%)	-	0.14 (0.04-0.52)	0.92 (0.78-1.0)

DLCO_corr_%	PAH versus control	< 70.3	100% (73.2%-99.3%)^a^	100% (85.7%-99.7%)^a^	-	-	1.0
	
	PAH versus ILD	Nil	-	-	-	-	0.42 (0.22-0.63)

FVC/DLCO	PAH versus control	≥ 1.66	64.3% (35.6%-86.0%)	96.7% (80.9%-99.8%)	19.3 (2.7- .)	0.37 (0.18-0.75)	0.90 (0.78-1.0)
	
	PAH versus ILD	≥ 1.82	50.0% (24.0%-76.0%)	94.4% (70.6%-99.7%)	9.0 (1.2-64.9)	0.53 (0.31-0.90)	0.71 (0.51-0.90)

DLCO < 70.3% and FVC/DLCO ≥ 1.82	PAH versus control	-	50.0% (24.0%-76.0%)	100% (85.9%-99.7%)	-	0.63 (0.43-0.91)	-
	
	PAH versus ILD	-	50.0% (24.0%-76.0%)	94.4% (70.6%-99.7%)	6.75 (0.9-50.2)	0.66 (0.45-0.98)	-

DLCO < 70.3% and FVC/DLCO ≥ 1.82 (A) and/or NT-proBNP ≥ 209.8 (B)	PAH versus control	-	100% (73.2%-99.3%)^a^	100% (85.9%-99.7%)^a^	-	-	-
	
	PAH versus ILD	-	100% (73.2%-99.3%)^a^	77.8% (51.9%-92.6%)	4.50 (1.90-10.7)	-	-

DLCO, diffusing capacity of lungs for carbon monoxide, % predicted; FVC, forced vital capacity, % predicted; ILD, interstitial lung disease; NT-proBNP, N-terminal pro brain natriuretic peptide; PAH, pulmonary arterial hypertension; (A) and (B) refer to the two components of the "composite" screening model. ^a^Because of the relatively small sample size, the upper limit of the 95% CI approximates but does not equal 100%.

### Prediction models and their properties

The results from the ROC curve analyses were used to form two screening models that were evaluated in PAH versus controls, and in PAH versus ILD patients (Table 3).

The first model (model 1) was based solely on PFTs. Here, patients who had DLCO_corr _< 70.3% and FVC/DLCO_corr _ratio ≥ 1.82 were regarded as having a "positive" screen. When applied to compare PAH with controls, this composite model yielded a sensitivity of 50.0% along with a specificity of 100% for PAH. When applied to compare PAH with ILD, the sensitivity remained at 50.0% with a specificity of 94.4%.

The second model (model 2) used a composite of DLCO_corr _< 70.3% with FVC/DLCO_corr _≥ 1.82 (component A) and NT-proBNP ≥ 209.8 pg/ml (component B). In this model, the screen was "negative" if both component A and component B were negative. The screen was positive if either component A and/or component B was positive. This model reliably distinguished PAH from controls with a sensitivity and specificity of 100%. When applied to PAH versus ILD groups, the screen yielded a sensitivity of 100% with a specificity of 77.8% (88% correct prediction).

### Model performance in an at-risk group

When the first model using only PFTs was applied to the at-risk group, 11 of 29 (37.9%) patients screened positive for the presence of PAH. Among these 11 patients, the mean sPAP_TTE _was 41.9 mm Hg (SD, 6.99).

When the second model was applied to the at-risk group, 17 of 29 (58.6%) patients screened positive for the presence of PAH. Among these 17 patients, mean sPAP_TTE _was 44.4 mm Hg (SD, 8.2).

The at-risk group had a subset of nine patients with a borderline RHC mPAP of 20 to 24 mm Hg who collectively had a mean sPAP_TTE _of 49.4 mm Hg (SD, 9.1). Of these nine patients, seven (77.7%) were screened positive. Of the remaining 20 patients in the at-risk group, who had not yet consented to RHC, 10 (50%) were screened positive, with a mean sPAP_TTE _of 41.4 mm Hg (SD, 4.6)

## Discussion

In this study, we showed that NT-proBNP, particularly when combined with PFTs, has the potential for use as a screening algorithm for PAH in patients with SSc, with "screen positive" patients then able to undergo further appropriate diagnostic testing. We also showed that NT-proBNP level correlates well with screening sPAP_TTE_, which is currently regarded as the most useful noninvasive method of screening for PAH. However, a number of important limitations of TTE are related to the lack of sufficient tricuspid regurgitation to estimate sPAP_TTE_, insufficient reliability in the context of coexistent lung disease, potential poor acoustic windows related to body habitus, the need for specific expertise in technique and interpretation, as well as issues related to cost and resource allocation [14,17]. It is against these limitations of echocardiography that NT-proBNP and PFTs in combination offer an accurate and more convenient "first tier" of screening tests for SSc patients.

**Figure 3:**
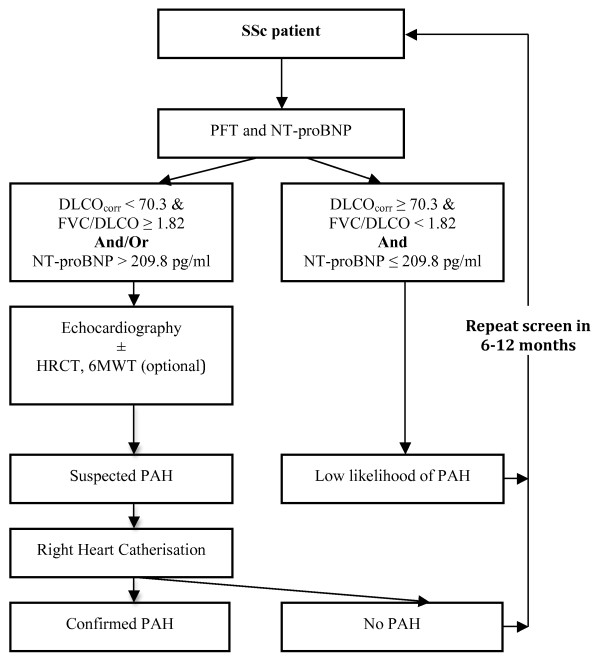
**A proposed screening algorithm for SSc-PAH**. 6MWT, 6-minute walk test; DLCO, diffusion capacity of lungs to carbon monoxide, percentage predicted; FVC, forced vital capacity, percentage predicted; HRCT, high-resolution computed tomography of lung; NT-proBNP, N-terminal pro-brain natriuretic peptide (pg/ml); PAH, pulmonary arterial hypertension; PFT, pulmonary-function test; SSc, systemic sclerosis.

In Figure 3, we have proposed a screening algorithm for SSc-PAH based on the findings of this study. In this screening algorithm, TTE is replaced by PFTs and NT-proBNP as the first tier of screening investigations. Thus only patients who have either DLCO_corr _< 70.3% with FVC/DLCO_corr _≥ 1.82, or NT-proBNP ≥ 209.8 pg/ml, or both, proceed to TTE, whereas those with DLCO_corr _≥ 70.3% and FVC/DLCO < 1.82 and NT-proBNP < 209.8 pg/ml are reassured and have repeated screening. If a patient is deemed to have a high clinical suggestion of PAH, then it would be appropriate to perform diagnostic tests to confirm or exclude PAH. The inclusion of PFTs (including FEV_1_, FVC, and DLCO) in the first tier of investigations also enables detection of patients with probable ILD who may require further investigation with HRCT.

Although we found that a sPAP_TTE _cut point of > 42 mm Hg had a 100% sensitivity and specificity for PAH, we are unable to recommend this diagnostic cut point, as the control group in our study were selected on the basis of a sPAP_TTE _< 30 mm Hg. In other studies, a lower cut-off point of sPAP_TTE _> 30 mm Hg had a sensitivity of 90%, whereas a higher cut-off point of sPAP_TTE _> 47 mm Hg had a specificity of 96% for PAH [15,17].

In this study, we demonstrated a correlation of NT-proBNP with key diagnostic and prognostic RHC parameters, including mPAP, mRAP, and PVR [20,25]. As RHC is the current gold-standard confirmatory investigation for PAH, these positive correlations again support the usefulness of NT-proBNP as a biomarker in SSc-PAH.

In the present study, we found a low or "normal" NT-proBNP level in all controls, but one patient with PAH had an NT-proBNP level below the cut point of 209.8 pg/ml. Such "false negatives" also were seen in a study from the Royal Free Hospital in the UK, which showed an NT-proBNP cut-point value of 395 pg/ml to have a sensitivity of 69% for PAH in a pilot study; this cut-point value was then separately tested in a larger prospective case-control study that produced a sensitivity of only 55.9% [20,25]. The reasons for these false negatives are unknown, but may include the interference of glycosylation with the NT-proBNP assay [26]. In our study, the patient with PAH and a low NT-proBNP had poor glycemic control. To overcome the potential problem of false negatives with NT-proBNP, we combined this biomarker with PFT. When combined with PFT, the NT-proBNP cut point of 209.8 pg/ml offered the excellent sensitivity (100%) for PAH that is desirable in a screening initiative. In the study by Mukerjee *et al*. [20], in which a higher NT-proBNP level of 395 pg/ml was determined as the cut point for PAH than in our study, 11 of 26 controls were undergoing the investigation for breathlessness, the cause of which may have led to a higher cut-point NT-proBNP level. In the subsequent validation study by Williams *et al*. [25], it is possible that the NT-proBNP cut point of 395 pg/ml was partly due to at least 20% of controls having ILD, which is in line with the findings from our study that suggest a higher NT-proBNP cut point (≥ 360.5 pg/ml) is required to separate PAH from ILD groups.

Our study has a number of strengths. The first is that all sera were assayed for NT-proBNP before the commencement of advanced therapies in a population newly diagnosed with PAH. Second, the inclusion of an ILD group offers a challenging and real-life complexity. Together, PAH and ILD account for more than 60% of SSc-related deaths, and the poorer prognosis of patients with ILD who also develop PAH is increasingly recognized [1,27]. Although a significantly higher mean NT-proBNP level was seen in the PAH group compared with the ILD group, a higher NT-proBNP cut point was required to separate the PAH group from the ILD group. This implies that ILD alone may result in modest elevation of NT-proBNP. These findings are consistent with those reported in studies of BNP that included SSc-ILD patients, suggesting modestly increased BNP in ILD patients without PAH [28]. Further, increased BNP levels are associated with greater mortality in ILD patients with coexistent PAH [28,29]. Alternatively, the higher cut point in the PAH-versus-ILD groups may reflect a degree of undiagnosed pulmonary hypertension and right ventricular compromise in the ILD group. For the purposes of screening, the addition of an FVC/DLCO cut point to the lower threshold value of NT-proBNP (209.8 pg/ml) incorporates an easy-to-calculate, inexpensive, and potentially useful clinical tool that can suggest the presence of pulmonary vascular disease.

The application of the screening model to the subgroup of at-risk patients with "borderline" pulmonary hypertension (that is, mPAP, 20 to 24 mm Hg, and PCWP, ≤ 15 mm Hg) who do not currently satisfy Dana Point criteria for PAH, revealed that a high proportion of these patients (77.7%) would have screened positive. Although the natural history of this group requires further longitudinal study, the high sensitivity of the algorithm in a group of patients that would be regarded as having abnormal pulmonary pressures, and possibly evolving PAH, is encouraging and is required of a screening tool [30].

Potential limitations of this pilot study may affect the generalizability of the findings. We have not included patients with left ventricular (LV) dysfunction, a factor that can increase the level of NT-proBNP. However, it is important to note that in our proposed screening algorithm, all patients with a positive NT-proBNP would progress to TTE to evaluate further for LV dysfunction and valvular heart disease. The goal of this screening algorithm is not to distinguish between PAH and left ventricular dysfunction but to select patients who may be developing cardiopulmonary complications for further investigations.

In this study, we also excluded patients with an eGFR < 30 ml/min, given the reduced renal excretion of NT-proBNP at these levels. However, with significant renal impairment, one would expect an increase in NT-proBNP, meaning a possible increase in false-positive screens but not an increase in false-negative screens. Further, this degree of renal compromise is not frequently seen in SSc patients.

Last, the study is limited by the relatively small size in each group and its observational case-control design. The prospective evaluation of this algorithm in a cohort of SSc patients is required to refine screening cut points, validate the model, assess predictive values, and determine the frequency of screening.

The cost-effectiveness of our proposed screening algorithm also merits evaluation. At present, the cost of TTE combined with PFT in Australia is approximately $A367, whereas the cost of the PFT combined with NT-proBNP assay is $A195. Therefore, potentially, our proposed screening algorithm may lead to a cost saving in screening for SSc-PAH, compared with the existing screening algorithm. Furthermore, NT-proBNP assays have become more clinically available with the widespread use of NT-proBNP in the diagnosis, prognosis, and risk stratification of patients with congestive cardiac failure.

## Conclusions

We propose the prospective validation of a screening algorithm for PAH that is considered positive if either NT-proBNP ≥ 209.8 pg/ml, or DLCO_corr _< 70.3% with FVC/DLCO_corr _≥ 1.82, or both. Patients who screen positive should be referred for TTE and considered for additional investigations, such as HRCT or 6MWT, and RHC, if appropriate.

## Abbreviations

6MWD: 6-minute walk distance; ANA: anti-nuclear antibody; anti-cent: anti-centromere antibody; anti-Scl70: anti- topoisomerase-1 antibody; ASCS: Australian Scleroderma Cohort Study; CO: cardiac output; dis. Duration: disease duration; DLCO_corr_: diffusion capacity of lung for carbon monoxide: corrected for hemoglobin; eGFR: estimated glomerular filtration rate; FEV_1_: forced expiratory volume in 1 second; FVC: forced vital capacity; FVC/DLCO: forced vital capacity/diffusion capacity of lung for carbon monoxide: corrected for hemoglobin; HRCT: high-resolution CT lung; ILD: interstitial lung disease; mPAP: mean pulmonary artery pressure; mRAP: mean right atrial pressure; NT-proBNP: N-terminal pro-brain natriuretic peptide; PAH: pulmonary arterial hypertension; PAP: pulmonary artery pressure; PCWP: pulmonary capillary wedge pressure; PFT: pulmonary-function test; PVR: pulmonary vascular resistance; RHC: right heart catherization; ROC: receiver operator characteristic; sPAP_TTE_: systolic pulmonary artery pressure; SSc-PAH: systemic sclerosis-related pulmonary arterial hypertension; TRV: tricuspid regurgitant velocity; TTE: transthoracic echocardiography; WHO-FC: World Health Organisation functional class.

## Competing interests

The authors declare that they have no competing interests.

## Authors' contributions

VT contributed to study design, collection and analysis of data, interpretation of results, and preparation of the manuscript. WS contributed to study design, collection of data, interpretation of results, and preparation of the manuscript. DP contributed to study design, interpretation of results, and preparation of the manuscript. OM contributed to collection of data, interpretation of results, and preparation of the manuscript. JB contributed to collection of data and preparation of the manuscript. DL contributed to study design, interpretation of results, and preparation of manuscript. KP contributed to collection of data and preparation of the manuscript. PH contributed to collection of data and preparation of the manuscript. JR contributed to the collection of data and preparation of the manuscript. JZ contributed to the collection of data and preparation of the manuscript. JS contributed to the collection of data and preparation of the manuscript. PN contributed to the collection of data and the preparation of the manuscript. KT contributed to the collection of data and preparation of the manuscript. DC contributed to interpretation of results and preparation of the manuscript. EG contributed to collection of data, interpretation of results, and preparation of the manuscript. PY contributed to collection of data, interpretation of results and preparation of the manuscript. SP contributed to the study design, collection of data, interpretation of results, and preparation of the manuscript. MN contributed to study design, collection and analysis of data, interpretation of results, and preparation of the manuscript. All authors read and approved the final manuscript.
